# Improving the visualization, interpretation and analysis of two-sample summary data Mendelian randomization via the Radial plot and Radial regression

**DOI:** 10.1093/ije/dyy265

**Published:** 2018-11-13

**Authors:** Jack Bowden, Wesley Spiller, Fabiola Del Greco M, Nuala Sheehan, John Thompson, Cosetta Minelli, George Davey Smith

First published online: 28 June 2018, *Int J Epidemiol*, doi: https://doi.org/10.1093/ije/dyy101

Since its initial publication, the article ‘Improving the visualization, interpretation and analysis of two-sample summary data Mendelian randomization via the Radial plot and Radial regression’ has been updated to include a funding statement relating to the authors Jack Bowden and George Davey Smith.

